# Harvard Odontological Society

**Published:** 1894-02

**Authors:** 

**Affiliations:** Harvard Odontological Society


### HARVARD ODONTOLOGICAL SOCIETY.

A MONTHLY meeting of the Harvard Odontological Society was held on Thursday evening, September 28, 1893, at Young's Hotel, Boston, with the President, Dr. Eddy, in the chair.

*President Eddy.*—It gives me great pleasure to announce that we are once more permitted to open our ranks to one of our former active fellows, and we shall surely give a hearty welcome to Dr. Charles H. Taft, of Chicago, who will read a paper entitled "Injurious Effects of Amalgam Fillings."

(For Dr. Taft's paper, see page 81.)

### DISCUSSION.

*Dr. Briggs.*—This is a question of great interest to us, and has been the subject of controversy for years. Though a physician myself, I have always recognized the position the general practitioner holds as being superior to my position as a specialist, and I have taken the stand towards amalgam fillings that if the family physicians believed it was not a good thing for their patients to have them, I was perfectly willing to take them out, at the same time declaring my own belief in the matter and feeling that they were perfectly safe.

This question, it seems to me, is a very hard one to decide. One has to begin by being a believer in the Hahnemannian theory. The question of this fine division of drugs is what the whole matter hinges on. If one accepts this theory, he must accept the fact that amalgam fillings are dangerous. Not being a follower of Hahnemann, I do not accept that theory. Then the question of proof comes in, and that is a very difficult thing to decide. Dr. Taft has seen cases where by taking out the fillings it has produced a cure. He does not exactly make the statement that amalgam fillings are poisonous,—only that in perhaps the majority of cases they interfere with drugs given according to Hahnemann's theory. My personal experience, and perhaps that of every dentist, is that I have seen no direct results from amalgam fillings,—that is, no cases in which I thought the amalgams in a patient's mouth have been doing any injury. I have been requested sometimes not to put them in, and have always acceded and followed that instruction. I have always been conservative in regard to amalgam, never having used it when I just as well could use some other material. Dr. Taft, when he went away, left behind him a great many cases where he had inserted amalgam fillings. It has been my privilege to see some of them since he has left, and I can only testify that he seems to have done great work in preserving teeth by the methods he followed.

What I would like to get at for my own edification and satisfaction is to prove that these things do the harm that is claimed, but it seems to me it is very hard for us to come to a point where we can meet on even ground, understanding as we do that it opens up the question of faith. I once asked a gentleman who was a chemist if any chemist did believe in Hahnemann. He said, "Yes;" and I asked him how they reconciled the two theories, and he said, "When they accept Hahnemann they drop their chemical knowledge and follow faith." That, of course, was a point of view from an unbeliever.

I would like to ask Dr. Taft what proof have we, other than in the cure of disease, that there is power in those high potencies of medicine, and when I say that I mean to put a great deal of stress on the one point, the *curing* of disease. Statistics show that disease is cured under all sorts of conditions. We know that the faith-cure, the Christian scientist, and any follower of any kind of medicine, can show a great many cures, and remarkable ones, and yet we are not prepared, when a woman says that she went to this Christian scientist, went sick and came away well,—we are hardly prepared to follow that particular creed. When we go to Montreal, in the cathedral there we hear stories of people who have come there lame and diseased and have thrown away their crutches and departed cured, but you would not ask us to believe that that is the best system of medicine to believe in. What I want to get at is the scientific solution of this question. I want enlightenment. I mean by that I would like to be convinced of anything that is going to help me to give my patients that service which is for their best interests, and we have men here to-night who seem to be fully equipped to explain this matter to us.

*Dr. Kimball.*—I do not wish to discuss the homœopathic law of cure, but simply to relate a few cases. In regard to chemistry, it may not be generally known that Hahnemann was one of the greatest chemists of his day, which fact ought to dispose of the question of the incompatibility of chemistry and homœopathy.

Speaking of the case that Dr. Taft detailed to some extent, the gentleman was a patient of mine. He had been under my care about a year and a half. His mouth was full of amalgam fillings. He was engaged in study at college and was constantly afflicted with sick headaches and seminal discharges at night, and was generally in a debilitated condition. I prescribed for him as carefully as I could from the symptoms presented, but the medicines did not seem to take effect. After the fillings were removed the change was marked and striking. He was able to work harder. He went through the Harvard school, where he was studying, very successfully and gained about twenty pounds in flesh, and now is able to do hospital work, night work, without any recurrence of his former trouble. This change, of course, cannot be placed to my credit alone, because the same remedies that were used before were used afterwards.

There was one case that I know of where no remedy was used at all, but the amalgam fillings were removed. The case was reported to me by a lady physician. A young girl, about eighteen years of age, came to her suffering from painful menstruation and a coryza during the menses. Formerly that function had been perfectly normal. She asked her how long that disturbance had been going on, and the reply was for several months. The physician was interested in this matter of amalgam fillings, and she asked the patient if she had any, and she said yes; some were put in about six months before, two months before the trouble began. She thought this would be a favorable opportunity to test the efficacy of removing them without giving any remedy, and told her to have the amalgam fillings removed and that she would not give her any medicine. There was no coryza the first month, but there was some pain; the second month there was no pain and no coryza, and there has been none since. That case is worth much more than cases in which remedies have been used.

Another case was one of Dr. Thurston's. I am sorry he is not able to be present to-night on account of sickness. The patient was a lady who was a great sufferer from nervous prostration and had been under the treatment of skilful physicians. Dr. Thurston noticed that she had a large number of amalgam fillings, which he advised her to have removed, and a marked change in her health was soon manifested, and she went on to a complete cure. I have one case of chronic nasal catarrh in which the removal of the amalgams has been of decided benefit, and indeed I am firmly of the opinion that chronic cases cannot be properly treated without removing the amalgams, and I am very unwilling to undertake a case without having them removed.

Of course we treat patients with amalgam fillings and get very good results, and in cases where we do not, it is possible that we may not prescribe correctly for them; at the same time, the effect of the mercury undoubtedly either has an injurious effect upon the system, as is evident in some cases, or it prevents the remedies from having the desired effect.

There was an extremely interesting case of a man who came from Australia and went to London and put himself under the care of an able physician there for a stomach trouble, a troublesome indigestion which had continued for years, and from which he could only obtain temporary relief. This man treated him for a year and admitted that he could do nothing for him, but he advised him to go to a certain physician in Brooklyn, who, he said, could cure him if any one could. He came over, and the Brooklyn physician, who tells the story, after hearing the history of the case, concluded there must be something operating as a bar to the cure. He examined his teeth and found a large number of amalgam fillings; he had them all removed, and immediately the man recovered from his trouble, and at the last accounts had had no return.

From these facts, I think if we meet with any difficulty in curing troubles of the throat or the stomach, diseases of the nervous system, painful menstruation and coryza, it would be well to see what effect it would have to remove the amalgam fillings.

*Dr. Briggs.*—If you will allow me just a minute, Mr. President? There is one thing that physicians do not and which every dentist does know, and that is that there is no work that is done in such a slovenly manner as the majority of work in amalgam. You will often find, after slovenly operators, overhanging fillings and fillings cut away so as to cause great local irritation, and do you always recognize that factor? You know what trouble you can get from the reflex action of a slight irritation, and if you would recognize the tremendous irritation that is going on in that mouth you could then account for any lack of effect from the medicine, and of course, if you take those fillings out and remove all that irritation, you would very naturally find an improvement. A great many times, I don't say in every case, you would find that the local irritation from these slovenly-made fillings would be the sole cause of troubles located in any part of the body.

*Dr. Kimball.*—The amalgams in the mouths that I examined seemed to be particularly well put in; the teeth seemed to be well cared for, and the patients were intelligent people who would be apt to notice local irritations.

*Dr. Briggs.*—Intelligent people do not always go to good dentists.

*Dr. Kennedy.*—I have been interested in what has been said thus far. I was much interested in the paper and confess the essayist introduced more homœopathy than I thought he would, in which, however, I am thankful to say I am interested. I like the sentiment voiced by Dr. Briggs, that men are ready to investigate. I believe that is right. Let us investigate, and if I understand it rightly, the purpose of Dr. Taft's paper is largely that it may stir you up to investigate for yourselves.

Now, I am not here to discuss the matter, much less to prove to you, or to demonstrate to you beyond any question, that injurious effects result from amalgam fillings. I simply come in here to testify to what I have observed, and I believe that is the thing to do. We should cultivate the habit of observation, for by it we learn many valuable truths. I do not think any less of a man because he differs from me, and I wouldn't give anything for a man who is not sceptical at times. One is very naturally sceptical concerning a thing of which he knows very little.

Now, there are two or three points here I want to refer to. In the first place, in regard to the treatment of chronic cases, where the treatment extends over a month or several months and sometimes a year. In speaking of this Dr. Taft says we are not to accept it because he says so, and I like that. I do not expect you to accept it because I assert it. I stand here and say that it is my conviction that amalgam fillings do affect some patients,—not all, or at least to that extent that we can observe it, but that it does decidedly affect some people I am most positive in my belief.

Dr. Briggs has said that he yields to the physician, and if the physician requests that amalgam fillings be removed, he acquiesces in that, and yet expresses his sceptical views on the subject. I have found a similar experience, as I have sent patients to dentists to have amalgam fillings removed, and the dentist would ask, "What crank have you for a physician? Of course, if he says so, I will do it."

I do not quite think that it is necessary to believe in homœopathy to become convinced that amalgam fillings are injurious. It is by observation that we can learn facts, and in order to give you some of the facts that I have observed and not prolong my remarks, I will mention a few cases that have come to my knowledge.

One was that of a clergyman who was troubled with his throat, which was very sensitive and repeatedly became inflamed, so much so that he was unable to go on with his professional work, and he spent thousands of dollars in this country and other countries trying to get rid of his trouble. Some one discovered that he had amalgam fillings in his mouth and advised their removal, and he recovered rapidly.

Another one, treated by a physician in this city, was that of a servant who had an ulcer on the inside of the cheek. The doctor gave her several prescriptions without any satisfactory result, and could not understand why his medicine had no effect. Noticing one day that directly opposite the ulcer was a tooth filled with amalgam, he said to her that she must have that filling removed. The servant went to a dentist, who removed the filling, and immediately she began to improve without further treatment.

Another case was that of a lady in this city,—an artist, who had been subject to attacks of sore throat, such as tonsillitis, laryngitis, and other affections, at frequent intervals extending over a period of twenty years. She came at length under the care of a physician who was a careful prescriber, but, do the best he could, he found there was something that prevented the action of the remedies,—certainly the treatment was very unsatisfactory in its results. Noticing that she had amalgam fillings, he told her that it might help her if she had them removed. She did so, and without any further treatment she began to recover from the feelings that she had thought were a part of her daily life. She has since that scarcely had a sore throat, and it is now a year and a half ago. I know the lady personally, and she will state the same to you at any time.

Another was a case of my own, of a lady who was troubled almost continuously with tonsillitis, pharyngitis, laryngitis, affections of the vocal chords, and more or less cough accompanying. I treated her for some time and studied her case faithfully. I would relieve her to a certain extent, but the trouble would soon return, and the results were very unsatisfactory. I then ordered the removal of some amalgam fillings. That was about three years ago, and I have given her but two or three prescriptions since. She removed about that time from Massachusetts to New Hampshire. Possibly the change may have resulted in this case from the difference in climate.

Last, but not least, at any rate not of the least interest to me, is my personal experience. I went last spring to my dentist and told him that I would like to have him remove the amalgam fillings in my teeth. He looked at them and said, "They are all right; they appear to be good and solid. What do you want them taken out for?" I replied, "I do not wish them in there. They may be all right, but I am satisfied they sometimes do harm in the mouths of patients. It is possible they may not affect me, but I wish them removed." So he took them out. To my surprise, within three weeks I began to notice a change in my digestion, which had troubled me for a dozen years, ever since I had had the amalgam fillings. After eating certain articles of food I would invariably have an attack, more or less severe, of what is commonly called heartburn,—a very disagreeable feeling. I have had very little since and have taken much less treatment for it. Furthermore, whenever I take a remedy now it acts far more promptly and effectively than formerly.

Now, these are points to which I am glad to bear testimony as the result of my observations. I do not expect that they will prove to you that amalgam fillings are injurious, but I hope they may tend to interest you to the extent that you will watch and investigate for yourselves.

*Dr. Hitchcock.*—Dr. Kennedy's remarks convince me that possibly I gave a patient a wrong treatment. A lady had two amalgam fillings in her mouth, and one of them, in the second upper molar, was loose, with redness about the gum and more or less coryza. According to the theory of the essayist, she may have been affected more or less dynamically by the filling, but after looking it over I decided that the inflammation of the gum was caused by the amalgam pressing against it, and was increased by her picking and trying to remove it. Now, from what has been said the filling should have been removed and some other material used, but the amalgam filling was simply trimmed, and there has been no trouble since.

Neither Dr. Taft nor Dr. Kennedy has told us what is the exact effect of mercury on the system. I have here the provings of mercurius; let me read: "The teeth feel loose, fall out. Toothache from caries or when the dentine is inflamed; returns in damp weather or evening air; pains tearing, lacerating, shooting into face and ears; worse from warmth of bed, from cold or warm things; better from rubbing the cheek. Gum painful to touch, swollen, receding from the teeth; edges whitish, bleeding; fetid odor from the mouth; ulcers with dark-red edges. Pulsating toothache worse at night; gum-boil."

"It is equally certain that Hahnemann himself admits the general aggravation of disease by homœopathic doses when administered in sensible quantities."

Now, to find out about this, let us give these medicines when there is reason to think that the patient is being affected by amalgam fillings,—it will do no harm. One thing surprises me,—namely, that gold is an antidote for mercury. How do the gentlemen get over this point? If gold is an antidote for mercury, then the gold fillings in the mouth would counteract the effect of the mercury, and the amalgam fillings would produce no ill effects.

I was talking at my home with a physician who laid more stress on the copper than on the mercury in amalgams. I find in looking over the Pharmacopœia that alum, arsenic, gold, belladonna, bismuth, borax, bromides, calcium, phosphorus, camphor, cannabis indica, cinchona, copper, sulphur, and many others produce provings on the teeth.

The way to prove this and find out who is right is for us to take a few of these remedies and try them. Then notice whether or not we get these mercurial provings. We can certainly get a larger dose of mercury into our system by mixing the amalgam in our hands than from a filling in the tooth; they have given us no examples of dentists being affected by it, though they are using amalgam almost daily. Why do we not produce these effects on all our patients by the mouth-mirror, which is coated with mercury?

*Dr. Allen.*—Those are old-fashioned mouth-mirrors, doctor; they are now lined with nitrate of silver.

*Dr. Hitchcock.*—That is just as bad. I have the provings right here. There are cases in which people are so sensitive to different metals that they cannot carry a silver or gold coin, or even a copper, in their pocket. To such a person coins will produce bad feelings in the head until removed from the pocket. You have heard of persons who when blindfolded can step on a coin and tell what kind of metal it is.

Why are we so afraid of putting silver fillings into a dead tooth? It seems to me that is the best treatment we could give.

Dr. Kennedy speaks of the indigestion which was relieved by the removal of the amalgam fillings. I have been troubled similarly, and have found relief by taking a little nux vomica, but there is no amalgam in my mouth.

*Dr. Kennedy.*—While having the amalgam fillings in my mouth the nux vomica didn't relieve me, but I have had occasion to take it since, and the relief could be noticed at once.

*Dr. Hitchcock.*—In speaking of a potency, you take one drop of the original drug and add ten drops of alcohol or water to it, then take one drop of the latter and add ten more, making one drop in a hundred for the second potency, do you not?

*Dr. Kimball.*—Potentation is a development of force. The original drop, by the process of shaking and mixing which it undergoes, medicates the whole one hundred drops; a drop of this medicates the next hundred, and so on. It is the same as with the magnet. You can take a magnet and magnetize one thousand magnets with it, and those magnets will become one thousand times as strong as the first one, and yet it will have lost none of its power.

*Dr. Hitchcock.*—That is not a parallel case, but isn't that the proportion, one drop in a hundred?

*Dr. Kimball.*—It is not the same as the dilution of a remedy. By this process the more you get rid of the material the more you develop the curative properties. The medicinal value is increased with every dilution.

*Dr. Hitchcock.*—For your general practice what is the highest potency given?

*Dr. Kimball.*—We find that we get the best results with medicines ranging from the thirtieth to the one-hundred-thousandth potency. Now, to show you that there is an efficacy even in the one-thousandth potency, you will permit me to relate an incident even if it does not pertain to this discussion.

A great many people are opposed to vaccination with the crude virus, and undoubtedly a great many children are killed with the best virus that can be obtained. Now, for an experiment, and I have done it twice within the last six months, once not later than three weeks ago, instead of vaccinating, I have given two powders of the one-thousandth potency of variolinum to a child ten years old, one at night and one in the morning, and within a week there were twenty umbilicated vesicles over its body, some as large as half the finger-nail, and when examined they exhibited the characteristics of the vaccine pustule. With such examples as this, we are compelled to think that there is some efficacy even in the high potencies.

*Dr. Hitchcock.*—I have been doing figuring a little, and find that the proportion of the original drug in the fifteenth dilution is one drop in 258,539,691 hogsheads.

*Dr. Smith.*—I assume that physicians know the manner in which these amalgam fillings are made, and I would like to ask what is their explanation of its effect upon the system? In the properly prepared amalgam filling a very small amount of mercury is used, and I would like to know how it affects the patient,—in what form?

*Dr. Stanton.*—I would suggest that Dr. Taft, being a dentist, could perhaps answer that question more intelligently.

*Dr. Taft.*—It gets into and affects the system in just the same way that the arsenic does in the wall-paper; you cannot tell just how that force, which is also a dynamic one, is doing its work upon one who spends considerable time in the room. That it does you have positive evidence in the unmistakable symptoms produced of arsenical poisoning. The question of potency in this matter is a very small one, and really cuts no figure in the point at issue. It is the dynamic action of the mercury that we have to consider, and knowing that it is such a deep-seated, deep-acting drug, we should be very careful how we expose our patients to its action.

I know it is very difficult for some men to accept the idea that there can be any symptoms produced upon a healthy person simulating symptoms of a disease from the administration of the one-hundred-thousandth or a much lower potency of a certain drug, but they can satisfy themselves of this fact by trying the different potencies on themselves or on other persons in health and noticing the effects. You will not have any difficulty in getting provings. If there is any law underlying the art of healing, the only way to get at it is to study the action of drugs upon a healthy person, and, as I suggested in the paper, if you will try these things for yourselves you will get all the evidence you care for.

*Dr. Smith.*—I think that the statistics, if you could get them, of the results obtained from the use of amalgam fillings would settle this question as to injurious effects. Amalgam may be guilty of all this poisoning, but it is not yet proved. And if we get dangerous effects from the small amount of mercury in the amalgam filling, why should we not expect it in the cement fillings, which also contain a powerful drug?

I will mention a case which might be claimed would tend to show an entirely opposite effect from amalgam fillings. A young lady in poor health had been in my hands a number of years. I had never used amalgam in the treatment of her teeth. I use but very little amalgam in my practice, and never in cases that I know are patients of homœopathic physicians. She was at that time under the treatment of a prominent homœopathic physician. Later she went on a visit to New York. What her trouble was of course I know not; and I think she was under the same treatment by the same physician while spending the winter in New York. In the summer she came into my hands again, and, much to my surprise, a dentist there had placed several amalgam fillings in her teeth, probably unknown to her; and so the lady for whom I had purposely avoided putting in amalgams came to me with these fillings in her mouth, but with her health perfectly restored. Now, if we can have a few more cases like that, cannot we just as well come to the conclusion that amalgam fillings are beneficial to the health? This is only one case that has come into my practice. Perhaps it would be unsafe, from the homœopathic stand-point, to experiment in this way, but as we seem to be in the line of experiments, would it not be well to put two or three amalgam fillings into the mouth of a person in poor health and under homœopathic treatment and see what the result would be? I do not see yet sufficient proof to warrant me in believing that amalgams are injurious. A majority of the people have amalgam fillings, and while I do not know the statistics, I fancy their general health will compare favorably with the richer class, who have mostly gold, oxyphosphate, or gutta-percha fillings, and few, if any, amalgam.

*Dr. Briggs.*—I spoke of the irritation of amalgam fillings purely from a mechanical point of view. Another source of irritation might be found in badly mixed amalgam fillings wearing away or being dissolved, especially in cases since the rage for copper amalgam came in. I cannot but feel that it is a very dangerous thing for copper to be taken into the system in quantities in such a mouth as that spoken of. If you intend to put them in, put them in and cover them with gold, or they will wash away and the salts of copper may be taken into the system. I would like to ask Dr. Taft if he considers that an amalgam filling covered with gold would have any power for harm in the mouth?

*Dr. Taft.*—That question has come up with me several times in my practice during the year, and I have discussed it with my colleagues as to whether there was a possibility of the system being affected by the mercury which might enter it through the tubuli. I feel that there hardly can be, and yet I consider it is safer not to use amalgam in combination fillings, even in crown cavities. You can cover the floor of a cavity with cement and then finish with gold just as easily, and it makes a better lining for the centre of the tooth than amalgam, and for myself I prefer not to take any chances on the amalgam.

I only want to speak of just one other case among the many that I have had this year. A young lady came to me from the dean of the college with which I am connected to have all her amalgams removed. She was a girl of very delicate health, had several large amalgams, and was troubled with chronic catarrh. She had not been improving in health at all, and upon advice of the dean I removed the fillings. She began to improve at once, and the next time she came she said she felt very much better in every way, and at a subsequent sitting there was not a trace of the catarrh left in her breath, which had previously been very offensive.

I have noticed during the past year a very steady and often rapid improvement, especially in patients troubled with chronic catarrh, chronic headaches, and chronic neuralgia, from whose mouths I have removed all amalgam fillings.

*Dr. Smith.*—The hour is getting late, and I would move that the discussion of this subject be extended to the next meeting.

Motion passed.

*Dr. Smith.*—I would further move that the Executive Committee be instructed to invite the homœopathic physicians who have been with us this evening, and others interested in this subject, to meet with us at our next meeting.

Carried.

The meeting was then adjourned.

Henry L. Upham, D.M.D.,
*Editor Harvard Odontological Society.*

